# Comparison of Cardiovascular Medicines Prices in Four European Countries

**DOI:** 10.3389/fpubh.2020.00433

**Published:** 2020-08-21

**Authors:** Zornitsa Mitkova, Mariya Vasileva, Alexandra Savova, Manoela Manova, Silvia Terezova, Guenka Petrova

**Affiliations:** ^1^Faculty of Pharmacy Bulgaria, Medical University of Sofia, Sofia, Bulgaria; ^2^National Council on Pricing and Reimbursement of Medicinal Products, Sofia, Bulgaria; ^3^Economics of Infrastructure Faculty, University of National and World Economy, Sofia, Bulgaria

**Keywords:** ACE- inhibitors, AT1-receptor blockers, manufacturer price, retail price, price difference

## Abstract

The aim of the current study was to compare pricing methodologies at the manufacturer, wholesale, and retail levels, and to estimate the price differences of AT1-receptor blockers (sartans), Angiotensin-converting enzyme (ACE)—inhibitors, and their fixed-dose combinations (FDCs) in four countries using similar methodologies: Slovakia, Greece, Bulgaria, and Romania (SK, GR, BG, and RO, respectively). The methodologies for manufacturer, wholesale, and retail price establishment have been compared using nationally implemented rules. Overlapping trademarks were established retrospectively on the manufacturer and retail levels in November 2017. The average price per tablet, percentage of price deviation, and statistically significant differences were calculated. The selected countries apply external reference pricing at the manufacturer level. A wide variation in the number of referent countries was observed (from 12 to 27). Despite the use of a regressive scale for price calculation, large variations between margins and value-added tax (VAT) are established, thus leading to different final medicine prices. This study found that medicine prices were lower in RO than in other selected countries. It was caused by the fact that 15 products had the lowest manufacturer price and 14 products had the lowest retail price in RO. Results of Kruskal–Wallis test showed that there were no significant differences between prices per tablet on the manufacturer and retail levels. In the group of fixed-dose combinations, ramipril/hydrochlorothiazide, and irbesartan/hydrochlorothiazide showed more than 100% deviation. The prices of cardiovascular medicines differed within the observed countries. The differences in pricing methodologies (e.g., margins, VAT) at the national level did not significantly affect retail prices, as a low manufacturer price usually leads to a low retail price.

## Introduction

Reference pricing is used as a method for price regulation in many European countries. Pharmaceuticals prices can be regulated, or can be a result of the market environment (1). The application of external reference pricing (ERP) started in the early the 90's (2), and is now the most commonly used price control measure in Europe (3). In each country, it is applied differently, usually in combination with other pharmaceutical policy measures. The size of the reference basket and the applied rules for a price calculation influence the price level and supply of medicines (4). Pricing criteria implemented in the CEE countries are similar and external reference pricing methodology is common in this region (5).

ERP is applied mainly to reimbursable medicines. The number of countries used as references ranges from three in Portugal to 30 in Poland. The pricing criteria used for medicinal products in the central and eastern European (CEE) countries are quite similar. Slovakia is the most frequently referenced country, whereas Cyprus, Iceland, Malta, Luxembourg, and Norway are not as frequently used as reference countries (6).

The methods for defining reference countries vary. Sometimes, the countries should be similar in terms of some characteristics, such as economic or geographical similarity and health system funding. Studies showed that at the national level, after ERP implementation, health expenditures decreased in the short term, as the prices are more likely to decline (7, 8). At the international level, ERP methodology can affect prices in other European countries, resulting in price fluctuations, delay launches, and manufacturers' withdrawal from markets where the price is low (9). Large launch delay to 3 years on average in Eastern Europe is found (10). Pharmaceutical pricing experts from Russia, Asia, Middle East, and South Africa confirm that ERP could be used for pricing decisions, but not as the only mechanism for price regulation (11).

Recent studies have shown that price differences exist across countries (12, 13). In countries with lower gross domestic product (GDP), where the resources are limited, the payers cannot effectively control the prices if ERP is used only as a cost containment measure (14). Studies show that the introduction of generic products with focus only on prices is not as effective as implementing other policies (15). The medicines are unaffordable for many European Union (EU) citizens. This may contribute to non-compliance, adherence, and rising direct and indirect costs (16–18).

In CEE countries, cardiovascular diseases (CVDs) prevalence is higher than in Western, Northern, and Southern European countries. The data showed that in the EU healthcare for CVDs is 53% (€111 billion), productivity loss is 26% (€54 billion), and informal care of people with CVDs is ~21% (€45 billion) of the total costs (19). Adequate access to cardiovascular (CV) medicines benefits the treatment of CVDs and could lead to decreased morbidity and mortality.

The main objectives of the study were as follows: [1] to explore the methodology of price calculation in four East European countries—Slovakia, Greece, Bulgaria, and Romania (SK, GR,BG, and RO, respectively) at the manufacturer, wholesale, and retail levels. [2] To compare prices between overlapping medicinal products from the therapeutic groups of AT1-receptor blockers (sartans), ACE inhibitor groups, and their fixed-dose combinations (FDCs) at the manufacturer and retail levels. [3] To calculate the price deviations from the lowest priced medicinal products to evaluate the overall effect of a market environment and country policy on medicine prices.

## Materials and Methods

Within the international cooperation for prices comparison EURIPID the RO, BG, GR, and SK are considered as countries that manage to maintain the lowest possible prices (https://www.euripid.eu/aboutus). This is one of the reasons for their selection (12, 20, 21). The second reason is that they all apply ERP for the regulation of medicine prices. The third reason is that they refer one to another and always are included in their national baskets despite the regulatory changes. The similar methodology for manufacturer price setting and variation in pricing methodology makes them an attractive for comparison.

The choice of therapeutic groups was determined by their importance in CVD therapy as a first-line recommended therapy in many guidelines (22, 23).

Comparative analysis between the methodologies for manufacturer and retail price establishment was performed among countries under consideration. Regulatory acts were used as a source of information and published scientific papers (24–27).

The manufacturer and retail prices of overlapping trademarks (produced by the same marketing authorization holder) of AT1-receptor blockers, ACE inhibitors, and their FDCs were collected from the official registers of the observed countries in November 2017 (28–31). Thirty-four overlapping trade names belonging to seven international non-proprietary names (INNs) and 9FDCsare considered for the price comparison analysis.

Price per tablet was calculated for each trademark. All prices were converted in Euro at the exchange rate of 1 euro = 1.956 BGN (Bulgarian Leva) and 1 euro =4.42 RON (Romanian Lei).

The average price per tablet was calculated at the manufacturer and retail levels between the four countries. The difference in the average and lowest prices was determined by deducting the lowest price from the average.

The statistical significance of price differences on the manufacturer and retail levels was examined using the Kruskal–Wallis test.

The percentage of deviation of medicines prices compared with the lowest one was calculated using the formula:

$$\begin{array}{c} Ip_{ij}=\frac{p_{ij}}{p_{imin}}\times 100\;\\ Ip_{ij}-price\;index\;of\;product\;i\;in\;a\;country\;j \end{array}$$

p_ij_ -manufacturer/retail price per tablet of the product i in a country j:

$$\begin{array}{l} p_{imin}-minimum\;price\;per\;tablet\;of\;the\;product\;i\;\\ PD_{ij}=Ip_{ij}-100\\ PD_{ij}-percentage\;price\;deviation\;of\;the\;product\;i\;in\;a\;country\;j \end{array}$$

Where,

Product i—compared INNs

Country j—selected countries.

## Results

### Comparison of Methods for Price Establishment

The selected countries applied ERP at the manufacturer level, and they refer their prices one to another. At the national level, there were differences and similarities in methodologies, thus leading to differences in the final prices of products (Table 1).

**Table 1:** Comparison of price establishment methodologies.

	**Slovakia**		**Greece**		**Romania**		**Bulgaria**	
Reference countries for calculation of manufacturer price	All EU countries (27)		All EU countries (27)		AT, BE, BG, CZ, DE, EL, ES, HU, IT, LT, PL, SK		Main countries: RO,FR, LT, LV, GR, SK,PT, IT, SI, ES ^*^Additional countries: BE, CZ, PL, HU, DK, FI, EE	
Pricing methodologies, applied for calculation of manufacturer price	The average of the three lowest prices of EU member states		The average of the three lowest prices of EU member states		The lowest ex-factory price for the same product out of 12 reference countries		The lowest ex-factory price for the same product out of 10 +7 reference countries	
Wholesale mark up *Wholesale margin (%) over manufacturer price, EUR*	Manufacturer price	Margin	Manufacturer price	Margin	Manufacturer price	Margin	Manufacturer price	Margin
	0.00–2.66	14.10%	Ex-factory price <200 euro	4.9%	0–11.31	14%	0–5 euro	7%
	2.67–5.31	11.10%	Ex-factory price >200 euro	1.5%	11.31–22.62	12%	5–15 euro	6%
	5.32–7.97	8.10%			22.62–67.87	10%	Over 15 euro	4% (but no more than 5 euro)
	7.98–13.28	5.10%			Over 67.87	6.78 euro		
	13.29–23.24	3.30%						
	23.25–39.83	2.70%						
	39.84–73.03	2.40%						
	73.04–165.97	2.25%						
	165.98–331.94	2.10%						
	331.95–663.88	1.95%						
	Over 663.88	1.80%						
Margins established for calculation of retail price	Wholesale price	Margin	Wholesale price	Margin	Wholesale price	Margin	Wholesale price	Margin
	0.00–2.66	32.90%	0–50	30.00%	0–5.66	24%	0–5 euro	20%
	2.67–5.31	25.90%	50.01–100	20.00%	5.66–11.31	20%	5–15 euro	18%
	5.32–7.97	18.90%	100.01–150	16.00%	11.31–22.62	16%	Over 15 euro	16% (but no more than 12.5euro)
	7.98–13.28	11.90%	150.01–200	14.00%	22.62 −67.87	12%		
	13.29–23.24	7.70%	200.01–300	12.00%	67.87	7.91 euro		
	23.25–39.83	6.30%	300.01–400	10.00%				
	39.84–73.03	5.60%	400.01–500	9.00%				
	73.04–165.97	5.25%	500.01–600	8.00%				
	165.98–331.94	4.90%	600.01–700	7.00%				
	331.95–663.88	4.55%	700.01–800	6.50%				
	Over 663.88	4.20%	800.01–900	6.00%				
			900.01–1,000	5.50%				
			1000.01–1,250	5.00%				
			1250.01–1,500	4.25%				
			1500.01–1,750	3.75%				
			1750.01–2,000	3.25%				
			2000.01–2,250	3.00%				
			2250.01–2,500	2.75%				
			2500.01–2,750	2.50%				
			2750.01–3,000	2.25%				
The mark ups are applied on the manufacturer price			Yes		No		No	Yes
VAT,%			10		6		9	20

In GR, the manufacturer prices are calculated as the average of the three lowest prices out of the other 27 EU countries in Europe. Since 2016, price revisions have been applied twice per year—May and November. Decisions for the inclusion of new products are made four times annually. The price of generic products was 65% of the price of the reference product (32) (Table 1).

SK applied reference pricing using prices from 27 European countries. The manufacturer price was calculated as the average of the lowest prices in three countries. In SK, the monthly ex-factory price of medicines was officially published, whereas reference price revisions were conducted every 6 months. The maximum price of the first generic product should not exceed 65% of the reference product price (5). Reimbursements list was revised four times annually (33) (Table 1).

In RO, a new pricing methodology has been applied since 2015. The ex-factory price should be the lowest from the 12 reference countries (where BG, SK, and GR are also included). The reference price of generics was 65% of the producer price for innovative drugs. The reference prices were updated once a year (in October) using the latest average exchange rate from RON to EUR (Table 1).

In BG, the ex-factory price may not be higher than the BGN equivalent of the lowest ex-factory price for the same medicinal product in the reference countries (the total number was 17: 10 main and 7 additional reference countries, Table 1). The Positive Drug List (PDL) was revised each month in terms of inclusion, changes, and/or exclusion of medicines. The manufacturer price of the generic products must not exceed 70% of the manufacturer price of the reference product included in the PDL (Table 1) (34).

### Comparison of CV Medicine Prices

The number of overlapping trade names and dosage forms of AT1-receptor blockers, ACE- inhibitors, and FDCs with the lowest prices at the manufacturer and retail levels are presented in Table 2. It is evident that the prices of medicines were lower in RO because 15 products had the lowest manufacturer price and 14 products have the lowest retail price in RO, followed by GR.

**Table 2:** Number of medicinal products with the lowest manufacturer and retail prices per tablet.

**Number of medicinal products with the lowest:**	**Country**			
	**SK**	**GR**	**RO**	**BG**
Manufacturer price per tablet, euro	4	9	15	6
Retail price per tablet. euro	7	12	14	1

The lowest and average price per tablet for each overlapping trademark was calculated as follows (Table 3):

**Table 3:** The lowest and average price per tablet on the manufacturer and retail levels.

**INN. dosage form**	**Manufacturer price (euro)**			**Difference between the average and the lowest price**	**Retail price (euro)**			**Difference between the average and the lowest price**
	**Country where the lowest price is found**	**The lowest manufacturer price per tablet, euro**	**Average manufacturer price per tablet, euro**		**Country where the lowest price is found**	**The lowest retail price per tablet, euro**	**Average retail price per tablet, euro**	
**ACE inhibitors and their FDCs**								
Perindopril 10 mg	GR	0.1497	0.1838	0.0341	GR	0.2163	0.2769	0.0606
Perindopril 5 mg	GR	0.1313	0.1456	0.0143	GR	0.1897	0.2216	0.0319
Ramipril 2.5 mg	RO	0.0243	0.0563	0.0320	RO	0.0374	0.0839	0.0465
Ramipril 5 mg	RO	0.0350	0.0812	0.0462	RO	0.0539	0.1218	0.0679
Ramipril 5 mg	RO	0.0538	0.1021	0.0483	RO	0.0829	0.1538	0.0709
Quinapril 20 mg	RO	0.0813	0.0998	0.0185	RO	0.1253	0.1519	0.0266
Zofenopril 30 mg	BG	0.2135	0.2223	0.0088	GR	0.3086	0.3310	0.0224
Perindopril/indapamide 2.5/0.625 mg	GR	0.1497	0.1705	0.0208	GR	0.2163	0.2570	0.0407
Perindopril/indapamide 5/1.25	GR	0.1510	0.1739	0.0229	GR	0.2183	0.2604	0.0421
Perindopril/indapamide 10/2.5 mg	RO	0.2587	0.2750	0.0163	RO	0.3857	0.4062	0.0205
Hydrochlorothiazide/ ramipril 5/25 mg	RO	0.0742	0.1263	0.0521	RO	0.1143	0.1901	0.0758
Quinapril/hydrochlorothiazide 20 mg/ 12.5 mg	SK	0.1296	0.1471	0.0175	SK	0.1842	0.2178	0.0336
Amlodipine/perindopril 10/10 mg	RO	0.2587	0.2685	0.0098	RO	0.3857	0.3975	0.0118
Amlodipine/perindopril 10/5 mg	BG	0.1773	0.2423	0.0650	GR	0.3830	0.3954	0.0124
Amlodipine/perindopril 5/10 mg	RO	0.1703	0.1832	0.0129	RO	0.2539	0.2740	0.0201
Amlodipine/indapamide/perindopril 10/2.5/10 mg	RO	0.3124	0.3300	0.0176	RO	0.4658	0.4849	0.0191
Amlodipine/indapamide/perindopril 10/2.5/5 mg	RO	0.2970	0.3114	0.0144	RO	0.4428	0.4586	0.0158
Amlodipine/indapamide/perindopril 5/1.25/10 mg	RO	0.2083	0.2264	0.0181	RO	0.3105	0.3363	0.0258
Amlodipine/indapamide/perindopril 5/1.25/5 mg	RO	0.1913	0.1990	0.0077	RO	0.2853	0.2968	0.0115
**AT1–receptor blockers (sartans) and their FDCs**								
Valsaratan 160 mg	SK	0.105	0.135	0.03	RO	0.173	0.212	0.039
Irbesartan 150 mg	RO	0.044	0.089	0.045	RO	0.067	0.133	0.066
Candesartan 16 mg	BG	0.109	0.134	0.025	BG	0.166	0.2	0.034
Telmisartan 80 mg	SK	0.111	0.136	0.025	SK	0.177	0.206	0.029
Telmisartan 80 mg	GR	0.216	0.233	0.017	GR	0.312	0.349	0.037
Irbesartan. HCTZ 300 mg/12.5 mg	GR	0.127	0.198	0.071	GR	0.184	0.301	0.117
Telmisartan/HCTZ 80 mg/12.5 mg	GR	0.158	0.159	0.001	GR	0.228	0.242	0.014
Telmisartan/HCTZ 80 mg/ 25 mg	RO	0.160	0.168	0.008	GR	0.232	0.256	0.024
Telmisartan/HCTZ 80 mg/12.5 mg	GR	0.243	0.269	0.026	GR	0.351	0.403	0.052
Telmisartan/ HCTZ 80 mg/ 25 mg	GR	0.247	0.249	0.002	GR	0.357	0.37	0.013
Amlodipín/ telmisartan 80 mg/10 mg	BG	0.514	0.539	0.025	BG	0.765	0.774	0.009
Amlodipín/ telmisartan 80 mg/5 mg	SK	0.52	0.539	0.019	SK	0.730	0.775	0.045
Valsartan/sakubitril 24 mg/26 mg	BG	2.09	2.256	0.166	SK	2.646	2.996	0.35
Valsartan/sakubitril 49 mg/51 mg	GR	2.092	2.296	0.204	SK	2.640	2.957	0.317
Valsartan/sakubitril 97 mg/103 mg	BG	2.09	2.256	0.166	SK	2.578	2.853	0.275

Four INNs of ACE inhibitors and 11 FDCs could be compared in all countries, and those are not the first in the class, such as enalapril. By comparing the manufacturer and retail prices of monoproducts in the group of ACE inhibitors, we confirmed that the unit prices were the lowest in RO, followed by GR and BG. The differences between the average and the lowest prices were not significant in terms of monetary cost. Regarding the FDCs, 1 product in RO (hydrochlorothiazide/ramipril 5/25 mg), 1 in SK (quinapril/ hydrochlorothiazide 20 mg/12.5 mg), and 2 in GR possessed the lowest manufacturer and retail prices (indapamide/perindopril 2.5/0.625 mg and indapamide/ perindopril 5/1.25 mg). In the group of AT-receptor blockers, comparison between 4 INNs and 10 FDCs was possible. At the manufacturer and retail levels, the lowest price was found in one product in RO (irbesartan 150 mg). The FDC GR had the lowest manufacturer price for telmisartan/hydrochlorothiazide 80 mg/12.5 mg, and the lowest retail price for irbesartan/hydrochlorothiazide 300/12.5 mg.

Results of Kruskal–Wallis test showed that there were no significant differences between the lowest and average price per tablet on the manufacturer and retail levels (*p* > 0.05).

Table 4 presents the rate of deviation in percentage from the lowest manufacturer and retail prices in each country (Table 4). Where there was only one product, the rate of deviation was not calculated, which happened often in RO (*n* = 14). This could mean that in RO, fewer products per INN were reimbursed. Higher rate of deviation in GR showed a wide competition per observed INN, which was most evident for ACE inhibitors.

**Table 4:** Percentage of deviation from the lowest manufacturer and retail price (%).

**INN. dosage (mg)**	**Percentage price deviation of the lowest manufacturer price per tablet in selected countries (%)**				**Percentage price deviation of the lowest retail price per tablet in selected countries (%)**			
	**SK**	**GR**	**RO**	**BG**	**SK**	**GR**	**RO**	**BG**
Perindopril 10 mg	13.1	–	64.9	13.1	22.4	–	70.2	19.4
Perindopril 5 mg	16.5	–	6.2	20.8	26.5	–	13.3	27.3
Ramipril 2.5 mg	32.9	458.4	–	34.6	39.8	424.1	–	32.9
Ramipril 5 mg	59.1	388.6	–	80	67	358.3	–	78.3
Ramipril 5 mg	47.4	264.3	–	47.2	54.8	241.4	–	45.7
Ramipril 20 mg	28.3	58.5	–	4.2	33	49.1	–	2.9
Zofenopril 30 mg	10.7	0.04	5.7	–	17.1	–	9	2.9
Perindopril/indapamide 2/0.625 mg^**^	13.1	–	29.3	13.1	22.4	–	33.5	19.4
Perindopril/indapamide 5/1.25 mg^**^	12.4	–	12.7	35.5	21.4	–	16.2	39.4
Indapamide/perindopril 10/2.5 mg^**^	14.9	6.2	–	4	14.3	3.1	–	3.8
Ramipril/hydrochlorothiazide 5/25 mg^*^	18.9	226.1	–	35.7	24.7	206.2	–	34.3
Quinapril/ HCTZ 20 mg/ 12.5 mg^*^	–	35.6	6.9	11.3	–	37.8	15.9	67.5
Amlodipín/perindopril 10/10 mg	7.3	3.7	–	4	7.7	0.7	–	3.8
Amlodipín/perindopril 10/5 mg	51.2	49.5	45.9	–	5.2	–	0.7	7
Amlodipín/perindopril 5/10 mg	8	6.1	–	16.2	12.8	2.9	–	16
Amlodipín/ind/perindopril 10/2.5/10 mg^**^	10.3	8.1	–	4.1	7.7	4.8	–	3.9
Amlodipín/ind/perindopril 10/2.5/5 mg^**^	8.5	6.7	–	4.1	6.8	3.5	–	3.9
Amlodipín/ind/perindopril 5/1.25/10 mg^**^	17.3	13.3	–	4.1	19.4	9.9	–	3.9
Amlodipín/ind/perindopril 5/1.25/5 mg^**^	6.6	5.2	–	4.2	10.2	2	–	4
Valsaratan 160 mg	–	68.8	7.3	38.3	15.6	47.6	–	27.4
Irbesartan 150 mg	68.1	240.4	–	111.3	64	218.9	–	109
Candesartan 16 mg	18.9	39.1	32.9	–	14.2	31.8	34.5	–
Telmisartan 80 mg	–	33.9	20.1	35.5	–	21.2	15.9	29.3
Telmisartan 80 mg	8.9	–	1.3	20.9	15.3	–	4.5	27.4
Irbesartan/HCTZ 300/12.5 mg^*^	41.1	–	11.2	169.3	52.6	–	18.6	184.1
Telmisartan/HCTZ 80/12.5 mg^*^	1.6	–	1.1	1.6	10.2	–	7.6	6.8
Telmisartan/HCTZ 80/ 25 mg^*^	17.9	0.4	–	3.6	26.7	–	6.1	8.6
Telmisartan/HCTZ 80/12.5 mg^*^	4.6	–	1.2	37.1	10	–	4.3	44.3
Telmisartan/HCTZ 80/25 mg^*^	3.4	0.5	–	0.9	8.1	–	2.6	3.4
Amlodipín/telmisartan 80/10mg	3.8	3.8	12.1	–	–	3.4	9.4	2.5
Amlodipín/telmisartan 80/5mg	–	0.1	9.9	4.5	–	3.1	11	11
Valsartan/sakubitril 24/26 mg	1.4	0.1	30.3	–	–	5.5	33.6	13.7
Valsartan/sakubitril 49/51 mg	1.4	–	30.2	7.5	–	2.2	23.2	22.7
Valsartan/sakubitril 97/103 mg	1.4	0.1	30.3	–	–	4.7	26.1	11.8

* HCTZ –hydrochlorothiazide;

** *indapamide; – indicated country with the lowest price*.

Within the group of monoproducts, the ACE inhibitor ramipril was the INN with a high price deviation of 241–458%, whereas in the group of AT-receptor antagonists, irbesartan had a deviation of 219–240%.

Between FDCs, prices of combinations of ramipril/hydrochlorothiazide in the group of ACE inhibitors and irbesartan/hydrochlorothiazide in the group of AT-receptor antagonists deviated by more than 100%.

## Discussion

The observed countries applied ERP as a methodology for the control of expenditures, but different calculation methods to compute the prices on a national level. The number of reference countries included in the basket varied from 12 to 27, but the lowest prices did not differ significantly, probably owing to simultaneous referencing at the manufacturing level. GR and SK used an average of three of the lowest prices, whereas BG and RO used the lowest prices from the countries in the basket. These differences in the number of referent countries, as well as the period of price revision, probably determined the differences between pharmaceuticals prices.

There are international collaborations for price comparison for the regulatory purposes as EURIPID for example but studying why some countries manage to maintain the lowest prices if beneficial for the international audience, especially for the medicines with high utilization (35). If the experience of those countries is analyzed and popularized, it will make important medicines affordable to many more citizens in Europe. This online database of EURIPID is currently exclusively available for national competent authorities for pricing and reimbursement of medicinal products that makes the scientific comparison of the methodologies and prices impossible via it.

The effectiveness of ERP measures is now widely discussed. The price revision in one country may contribute to changes in the others. A literature review showed that ERP application as a policy measure depends mainly on implementation and rules within the countries (36, 37). ERP is a well-known and widely used tool to control expenditures, but price control should be used together with other policies, especially those supporting the rational use of medicines and improving prescribing behavior (38).

A systematic review proved that reference pricing reduces pharmaceutical prices and, hence, expenditures and leads to substitution toward lower-priced drugs. This study also confirmed the need for new effective pricing policies, including value-based pricing, managed entry agreements, and health technology assessment (HTA) (39). At the analysis, BG and RO use managed entry agreements (40), whereas HTA is performed in BG, RO, and SK.

Over 2,000 drugs have disappeared from the market in the last 5 years, thus affecting patients' access to therapy and enhancing parallel exports (41, 42). Such a negative tendency can ruin the concept of ERP, which is why such studies are necessary.

A simulation of pharmaceutical prices showed a 15% reduction over 10 years. More detailed country baskets and frequent price revisions lead to higher price reductions. Revision frequencies also varied and contribute to price divergence and international price decrease (43).

The frequent price revision in BG probably led to a high rate of prices decreasing between 4 and 75.4% (44, 45). In GR, an average price decrease of 9.5% was achieved after the changes in the reference price system in 2010. Prices of ~12,000 medicinal products were recalculated because of the new system introduced in 2010 (46). Nevertheless, the study found the most significant deviation (more than 100%) for the six products in GR, thus confirming that other factors, such as the company's policy, taxes, and country environment, also affected the final medicine prices.

The SK new reference pricing system, which was introduced in 2012, was expected to create savings estimated at € 75 million. However, in SK, ERP resulted in higher prices compared with countries with similar income levels owing to the selection of reference countries (8).

A previous study confirmed that there is no substantial reduction in international price differences within EU countries applying EPR (47). We found that a small difference existed between the average and lowest prices, and there was a lack of significant difference in the same trademarks. However, after applying the price deviation approach, we observed large variations in prices between some of the countries. Therefore, the use of different approaches and points of view could provide more comprehensive and objective data of the existing price differences. It could be used by manufacturers and regulatory bodies when determining a price variation between countries. It is also important for receiving information on CV medicine utilization and how it is affected by price variation, a problem for which there is relatively limited information in the country (48).

The observed price deviation varied from 0.1 to 458%, thus confirming that the existing magnitude of the price difference was significant as a value. However, only six medicinal products showed more than 100% deviation from the lowest price. ACE inhibitor prices revealed the highest hesitations, whereas FDCs prices did not differ at such high degrees. Likewise, there were no products found at the same price in two or more countries. The reasons for such variance were probably more related to the health insurance environments and the country policy (49).

A price comparison of high-cost originator medicines in some European countries found lower prices in GR, Hungary, SK, and the UK. German and Swedish, Danish, and Irish prices were found at the upper end (7). Another study showed the highest prices in Germany (9), whereas one of the lowest prices is found in RO. Our study also found the lowest prices of CV medicines in RO (at the manufacturer and retail levels), despite the fact that the list of reimbursed medicines in RO was not updated between 2008 and 2015. Other factors, such as margins, VAT, or exchange rate, lead to low medicine prices.

A previous study on CV medicine prices showed that BG and RO follow the same methodology, but the differences in VAT and margins set different retail prices. The lower wholesale and retail margins in BG lead to a lower retail price, regardless of the higher VAT (20% in BG and 9% in RO). Therefore, the VAT influence is not the one factor for final retail price formation (50). The lower VAT rate has been balanced with higher margins in some countries.

The study found that differences between the prices of CV medicines existed, despite the expectation that ERP would equalize and reduce them. The differences between retail prices in the four countries under consideration were higher than those found at the manufacturer level. This was due to the established mechanisms for calculation of final medicine prices and ERP applications.

Our study confirms ERP methodology limitations and establishes price difference in reference countries, if they are compared simultaneously. This is the first study comparing prices of CV -medicines on manufacturer and retail level and provides direct data for ERP and implemented methodologies influence on final prices. The price comparison is widely discussed issue and often used from pharmaceutical companies for decision of product launches, from regulatory bodies for amendment of legislation or from other researchers reporting implemented regulatory measures for price control. The limitation of the study is a small number of overlapping trade names found (on total 34 in four countries) from overall variety of medicinal products approved in EU. This prompts the necessity for further studies exploring price differences and ERP influence on total medicines market.

Overall, the study found that lower manufacturer prices led to lower retail prices. The deviation in prices revealed their sensitivity to health policies and the market environment. It also contributed to manufacturers' decisions and therapeutic competition within a country.

In summary, the prices of CV medicines differed within the observed countries. The differences in pricing methodologies (e.g., margins, VAT) at the national level could not influence retail prices significantly, as a low manufacturer price usually led to a low retail price. From our results, we can conclude that RO was the country with the lowest prices of CV medicines (AT-receptor blockers and ACE inhibitors), followed by GR owing to financial crisis and low incomes. It could favor patients' affordability and cardiovascular therapy in those countries. Although BG had very frequent price revision as well as the lowest GDP per capita in the EU, this was not the key driver for lower prices, especially at the retail level.

## Data Availability Statement

The datasets presented in this study can be found in online repositories. The names of the repository/repositories and accession number(s) can be found below: National Council on prices and reimbursement of medicinal products. Registers PDL. Annex 1 https://portal.ncpr.bg/registers/pages/register/archive.xhtml Ministerstvo zdravotníctva; Available at: http://www.health.gov.sk/?kategorizacia-a-ceny Yπoϵργεí Yγεíας/Ministry of health; Available at: http://www.moh.gov.gr/articles/times-farmakwn/deltia-timwn Casa Nationalâ de Asigurâri de Sânâtata/National Health Insurance Fund Available at: http://www.cnas.ro/category/lista-medicamentelor.html.

## Author Contributions

GP conceived and designed the investigation and collected the data. ZM, AS, and MM prepared medicine selection and price calculations. MV performed a methodology comparison. ST and MM analyzed the data and performed the deviation percentage analysis. ZM performed the statistical analysis. GP designs the result interpretation and discussion. All authors wrote and revised the manuscript, and approved its submission for publication, confirming that that the work is original.

## Conflict of Interest

The authors declare that the research was conducted in the absence of any commercial or financial relationships that could be construed as a potential conflict of interest.

## Abbreviations

ACE, inhibitors: Angiotensin-converting enzyme inhibitors
AT1, receptor blockers, sartans: Angiotensin II type 1 receptor blockers
CEE, countries: Central and Eastern European countries
CVD: cardiovascular diseases
ERP: external reference pricing
EU: European Union
FDCs: fixed-dose combinations
INN: International non-proprietary name
PDL: Positive Drug List
VAT: value-added tax.
